# First trimester of pregnancy TSH laboratory specific reference intervals established by an indirect method

**DOI:** 10.11613/BM.2026.010704

**Published:** 2025-12-15

**Authors:** María Sanz-Felisi, Ariadna Arbiol-Roca, Paula Sánchez-García, Alicia Madurga

**Affiliations:** 1 Clinical Laboratory, Bellvitge University Hospital, L’Hospitalet de Llobregat, Barcelona, Spain; 2 Institut Català de la Salut, Atenció a la salut sexual i reproductiva Torrassa, Barcelona, Spain

**Keywords:** indirect method, pregnant women, reference intervals, thyroid stimulating hormone

## Abstract

**Introduction:**

This study established laboratory and trimester specific indirect reference intervals (RIs) for thyroid stimulating hormone (TSH).

**Materials and methods:**

A retrospective observational study was performed at a tertiary-care laboratory’s hospital during 12 months. Between February 2023 and February 2024, TSH results from 2166 women in their first trimester of pregnancy were retrieved. Only outpatients coming from primary care were included in the study. After applying exclusion and outlier criteria, TSH results from 1300 patients were analyzed to establish new RIs using the 2.5th and 97.5th percentiles by the non-parametric percentile method. These RIs were verified by an indirect method analyzing 486 TSH results from a cohort of pregnant women that were extracted from April to June 2024, and a direct prospective study of 28 pregnant women from a primary care center. All TSH tests were measured using a Cobas 8000 e801 system (Roche, Basel, Switzerland).

**Results:**

The TSH RIs were 0.60-4.33 mIU/L. Both verification methods met the requirements of the CLSI guidelines.

**Conclusions:**

The indirect method could be used to establish and verify local RIs for TSH in first trimester pregnant women. This may reduce misclassification of pregnant women undergoing thyroid function tests.

## Graphical abstract

**Figure:**
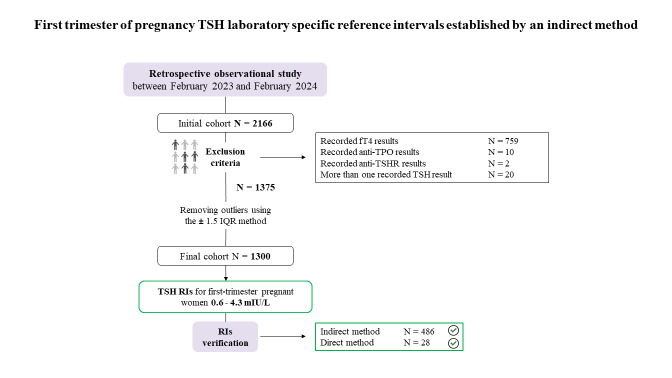

## Highlights

• The upper reference limit of the study for thyroid stimulating hormone (TSH) using the Roche Elecsys TSH reagent was 4.33 mIU/L

• The new reference intervals (RIs) are consistent with previous publications and the 2017 ATA guideline recommendation

• The RIs could reduce the risk of misdiagnosis and optimize thyroid management during pregnancy

## Introduction

Thyroid hormones play a vital role during pregnancy, influencing maternal wellbeing and fetal development. Maternal hypothyroidism is consistently associated with an increased risk of adverse pregnancy outcomes, including premature birth, low birth weight, pregnancy loss, and reduced intelligence quotient in offspring. Additionally, this condition can adversely affect the cognitive and neurological development of the fetus (*1*, *2*). Hence, a precise evaluation of the thyroid function during pregnancy and prompt intervention when required are crucial for correct fetal development. Considering these premises, thyroid stimulating hormone (TSH) is necessary for diagnosing thyroid disorders.

During pregnancy, particularly in the first trimester, several physiological changes influence thyroid function. The structural similarity between human chorionic gonadotropin (hCG) and TSH causes hCG to stimulate thyroid hormone production, which can reduce TSH concentrations. Elevated estrogen concentrations also increase thyroid-binding globulin (TBG), which binds to thyroid hormones and reduces their blood concentrations. This in turn triggers the secretion of TSH and promotes further production of thyroid hormones. It is also important to consider that normal pregnancy is associated with an increased need for iodine and increased renal excretion of this element (*1*, *2*).

Therefore, TSH results of healthy pregnant women differ from those of healthy non-pregnant women, and for this reason the American Thyroid Association (ATA) postulates the need for trimester specific reference intervals (RIs) for serum TSH based on the local population (*3*, *4*). These RIs should be based on pregnant women with no known thyroid disease, optimal iodine intake, and negative anti-thyroperoxidase antibody (anti-TPO) status.

The establishment and verification of RIs using conventional direct methods are often complicated by resource limitations or specific patient demographics (*5*). Jones *et al.*, from the working group of the IFCC Committee on Reference Intervals and Decision Limits (C-RIDL), aims to promote the adoption of indirect methods for establishing and validating RIs to support the development of improved statistical techniques for these studies (*6*).

Different laboratories may use different immunoassays to measure TSH, which can lead to discrepancies between TSH RIs. For these reasons, it is important to establish laboratory specific assay RIs to ensure consistency and reliability of test results (*7*, *9*).

The aim of this study was to establish first trimester TSH RIs using an indirect method and the Elecsys TSH Roche Diagnostics reagent (Roche, Basel, Switzerland).

## Materials and methods

### Study design

A retrospective observational study was conducted over 12 months (February 2023 to February 2024) at Bellvitge University Hospital. This 700-bed tertiary care teaching hospital is located in L’Hospitalet de Llobregat, Barcelona, Spain, and receives an average of 4000 laboratory requests *per* day.

Laboratory results for TSH of pregnant women on request for the first trimester protocol were obtained from the Laboratory Information System (LIS) (Modulab, Werfen, Barcelona, Spain). A total of 2166 TSH results were extracted from the LIS. Only outpatients coming from primary care were included in the study.

The analysis of the first trimester of pregnancy corresponds to a gestational age between 8 and 13 weeks and 6 days (*10*).

Exclusion criteria for this study included patients with recorded serum concentrations of free thyroxine (fT4), free triiodothyronine (fT3), anti-TPO, anti-thyroglobulin (anti-Tg) and/or anti-thyrotropin receptor (anti-TSHR) antibodies because our hospital’s laboratory protocols state that the LIS automatically adds free thyroid hormone and thyroid antibody measurements when TSH values fall outside the RIs.

Patients were also excluded from the study if they had follow-up thyroid function tests or a previous diagnosis related to thyroid function. The medical history and current treatment of all patients with TSH values outside the existing RIs were reviewed.

Data on TSH values, maternal age, ethnicity, weight, and type of pregnancy were extracted from the LIS and the SsdwLab program (SBP Soft, Girona, Spain).

The study protocol received approval from the institutional Clinical Research Ethics Committee (PR144/24). In regards to the direct verification study, each patient was given detailed information and provided written informed consent before blood sample collection. Patients’ clinical histories were reviewed using the hospital’s Physical Examination Information System (SAP GUI v.770, SAP SE, Walldorf, Germany).

To verify the RIs, two different studies were performed. The first one was a retrospective observational study based on the indirect method. A new cohort with the same characteristics as the population used to establish the RIs was selected. Another data export was performed from the LIS between April and June 2024. A total of 1391 TSH results were collected from the LIS, which were reduced to 486 after the exclusion criteria were applied. The RIs were verified using the same exclusion criteria as in the first study.

The direct method was used for the second verification of the study. A prospective study involving 28 first trimester pregnant patients recruited from a primary care women’s sexual and reproductive health center was performed. Patients were selected from the laboratory based on their medical history. A midwife nurse was responsible for recruitment and obtaining informed consent. Inclusion criteria for the selection of patients were healthy, pregnant women with normal pregnancies and no known complications. Exclusion criteria were a personal or family history of thyroid disease or other autoimmune diseases, previous treatment with thyroid medication or iodine irradiation, a history of recurrent miscarriage, and an age of over 37 years.

### Methods

Each venous blood sample was collected into 8.5 mL serum tubes with separator gel and clot activator (Becton Dickinson (BD) Vacutainer SSTTM II Advance, Reference No. 366468). The time of blood collection was performed in the morning between 8 and 9 a.m. After 30 minutes of clotting time, the tubes were centrifuged at 1500xg for 10 minutes at 20-25 ºC and subsequently analysed.

All analytes were measured using the electrochemiluminescence immunoassay analyzer Cobas 8000 (Roche, Basel, Switzerland). TSH serum concentration was measured using the Elecsys TSH reagent (Roche, Basel, Switzerland). The laboratory is accredited to UNE/EN ISO 15189:2023. Internal and external quality control achieved the metrological requirements of the laboratory during the study period.

### Statistical analysis

The distribution of TSH serum concentration was assessed for normality using the Shapiro-Wilk test, and a histogram was also used for the visual assessment. Logarithmic transformation was used to adjust for data distribution. To remove outliers, the interquartile range (IQR) was calculated using the first quartile Q1 (1.19) and the third quartile Q3 (2.65). After removing outliers using the ± 1.5 IQR method, RIs for TSH serum concentration were calculated by the non-parametric percentile method, using the original data, according to the CLSI guidelines C28-A3. Reference intervals with 90% of confidence intervals (CIs) were defined by the 2.5th and 97.5th percentiles of the distribution of TSH results in the cohort without outliers.

Data management and statistical analyses were conducted using the Stata Statistical Software: Release 14 (Stata Corp LP, College Station, TX, USA) and the Analyse-it software package for Excel 2016 (Analyse-it Software, Ltd., Leeds, UK).

## Results

**Figure 1:**
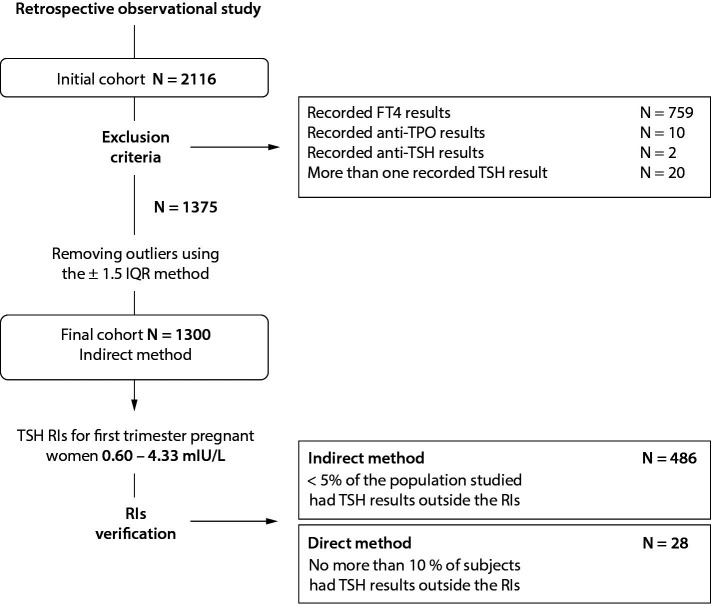
Flowchart for cohort selection and reference intervals verification using an indirect method. LIS - laboratory information system. TSH - thyroid stimulating hormone. fT4 - free thyroxin. fT3 - free triiodothyronine. anti-TPO - anti-thyroperoxidase antibodies. anti-Tg - anti-thyroglobulin antibodies. anti-TSHR - anti-thyrotropin receptor antibodies. RIs - reference intervals.

**Figure 2:**
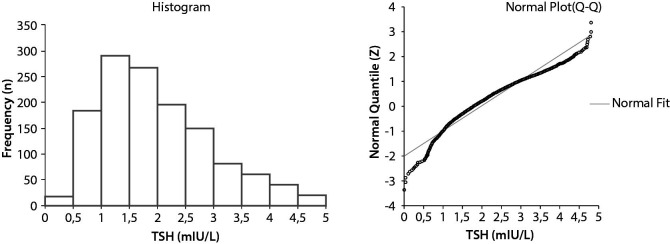
Histogram of the data distribution and normality Q-Q plot. TSH - thyroid stimulating hormone.

From the initial data of 2166 TSH results obtained from the LIS, 1375 TSH results were included after applying exclusion criteria (Figure 1). After removing the outliers and applying 1.5 IQR, the final data included 1300 TSH results. The data did not fit a Gaussian distribution for either raw or log-transformed data (Figure 2).

The median TSH serum concentration was 1.76 mIU/L (0.01-4.81 mIU/L) and the median maternal age was 32 years (16-38 years). The majority of patients were of Caucasian ethnicity (75%) and had a singleton pregnancy (98%). The median weight was 65 kg (31-157 kg).

The obtained TSH RIs with 90% CIs of first trimester pregnant women were: 0.60 (0.56 to 0.62)-4.33 (4.19 to 4.44) mIU/L.

The newly established RIs were validated using an indirect method. The data contained 486 results, and the median TSH serum concentration was 1.65 mIU/L (0.01-5.43 mIU/L). The verification was considered valid if less than 5% of the results fell outside the RIs. Only 17 patients (3.5%) had TSH results outside the new RIs that did not exceed the 5% requirement, confirming the validity of the new RIs (*11*).

Finally, the RIs were verified using a direct method (*12*). According to the CLSI EP28-A3 guideline, the RIs were deemed valid if no more than two of the 20 tested subjects’ values (or 10% of the test results) fell outside the reported RIs (*11*). A total of 28 healthy pregnant women were recruited from a primary care centre. The median TSH serum concentration was 2.16 mIU/L (0.23-4.71 mIU/L). Only two patients had a result below and above the interval limits (0.60-4.33 mIU/L). This means that 2/28 of the TSH results were outside the new RIs and therefore in compliance with the CLSI requirements (*11*).

## Discussion

In this study, we estimated RIs for serum TSH (0.60-4.33 mIU/L) in pregnant women during the first trimester of pregnancy using an indirect approach. This method has been shown to be effective in identifying clinically significant changes in analyte concentrations that might otherwise go undetected (*13*-*18*).

According to the 2011 ATA guideline, the upper reference limit TSH serum concentration during pregnancy was established at 2.50 mIU/L for the first trimester of pregnancy (*19*). It has previously been reported that this universal cut-off is unhelpful and unrealistically low in many situations, creating a false sense of simplicity in a complex situation (*3*).

Following this publication, larger cohort studies have produced center specific and trimester specific RIs during pregnancy. Studies of pregnant women in Asia, India, and the Netherlands have shown significantly higher TSH RIs for each trimester of pregnancy (*20*-*23*). For instance, Marwaha *et al.*, found that TSH RIs in a healthy Indian population were 0.60-5.00 mIU/L in the first trimester, using the direct method (*21*).

The ATA released an updated guideline in 2017 (*4*). This guideline recommends using an upper reference limit of approximately 4.00 mIU/L in the absence of internal or transferable pregnancy specific TSH RIs.

It should be noted that TSH RIs for different methods and reagent manufacturers might vary as they have been established using male and non-pregnant women and different antibodies. The decrease in serum TSH due to high concentrations of hCG during pregnancy should also be taken into consideration (*23*).

The manufacturer also provided RIs for pregnant women during their first trimester (0.33-4.59 mIU/L) and for females in different age groups (1.01-5.09 mIU/L for 11-20 years old; 0.44-3.63 mIU/L for 20-39 years old; and 0.16-3.94 mIU/L for 40-69 years old), based on various German studies (*24*, *25*).

McNeil and Stanford claimed that a number of recent studies had shown that a more realistic RI was between 3.00 and 4.00 mIU/L, depending on the analytical method used (*3*). In their review, they also reported an average first trimester 97.5th percentile for TSH of 4.00 mIU/L when using Roche analysers (*3*).

Following the ATA recommendations we have established specific TSH RIs for pregnant women during the first trimester of pregnancy (*4*). The prior TSH RIs used in our laboratory were based on a national, cross-sectional, population survey of individual’s ≥ 18 years old (0.57-5.51 mUI/L) (*26*).

The results of this study are consistent with the aforementioned, as the upper RI is 4.33 mIU/L, very close to the 4 mIU/L proposed by McNeil and Stanford (*3*). These RIs are also in accordance with the RIs proposed by the manufacturer for pregnant women during the first trimester, and lower than the ones reported for non-pregnant women, which is consistent with the downward shift in TSH during the early stages of pregnancy (*24*).

In a 2025 study, Sun *et al.* used a Siemens ADVIA Centaur XP analyzer to calculate the TSH RIs for pregnant women in a Chinese population (*27*). Their results showed lower RIs in the first trimester of pregnancy (0.02-3.39 mIU/L) than those observed in this study. This exemplifies the influence of different populations and analytical methods, and the importance of establishing laboratory-specific TSH RIs.

Recently, Dorizzi *et al.*, calculated TSH RIs (0.34-3.81 mIU/L) for pregnant women by the direct method (*28*). Their study was focused on a different time setting, since it was performed at 14-16 weeks of gestation, whereas ours was performed at 8-13 weeks. They also evaluated iodine intake by measuring urinary iodine excretion, concluding that mild and moderate iodine deficiencies did not affect the RIs (*28*).

Thus, this study incorporates and builds on previous recommendations in compliance with the CLSI guidelines to improve the accuracy and applicability of RIs using the indirect method (*11*, *13*-*18*). This data is also consistent with reports using the direct method.

Furthermore, the RIs were also verified using a direct method that relies on a well-characterized reference population, as well as standardized sample collection and analysis. By contrast, the RIs were also verified using an indirect method involving retrospective data and statistical filtering, reflecting the actual analytical and pre-analytical conditions of the laboratory’s patient population. Consequently, both verification methods offer complementary value to the process (*8*).

Although it is presumed that our population’s iodine intake is adequate, and evaluating iodine status is not mandatory in areas without iodine deficiencies, a limitation of our study was that the subjects’ iodine status was not measured (*29*, *30*).

Recently, Osinga *et al.* did a systematic revision and meta-analysis, and concluded that not excluding TPOAb-positive individuals led to an increase in the upper limit of TSH RI in all cohorts, with a mean increase of 0.65 (0.05-1.34) mIU/L in the first trimester of pregnancy (*31*). In this regard, indirect methods require robust statistical methods to differentiate between normal and pathological results. Another limitation of the present study is that we intentionally excluded all patients who had undergone more than one thyroid hormone and/or anti-thyroid antibody test. This was done to ensure consistency in the dataset and to prevent individuals with thyroid disease from being selected.

In conclusion, the results of this study highlight the importance of establishing accurate, population specific RIs for TSH in the first trimester of pregnancy. It is a robust and comprehensive approach that takes into account the population characteristics, the laboratory methodology, and preanalytical factors such as the time at which blood was collected.

## Data Availability

The data generated and analyzed in the presented study are not publicly available due to preserve individuals’ privacy under the European General Data Protection Regulation, but are available from the corresponding author on request.

## References

[1] Casey BM Dashe JS Wells CE McIntire D Byrd W Leveno KJ Subclinical hypothyroidism and pregnancy outcomes. Obstet Gynecol. 2005;105:239–45. 10.1097/01.AOG.0000152345.99421.22 15684146

[2] Salazar P Cisternas P Martinez M Inestrosa NC. Hypothyroidism and cognitive disorders during development and adulthood: Implications in the central nervous system. Mol Neurobiol. 2019;56:2952–63. 10.1007/s12035-018-1270-y 30073507

[3] McNeil AR Stanford PE. Reporting thyroid function tests in pregnancy. Clin Biochem Rev. 2015;36:109–26. 26900190 PMC4758281

[4] Alexander EK Pearce EN Brent GA Brown RS Chen H Dosiou C 2017 guidelines of the American Thyroid Association for the diagnosis and management of thyroid disease during pregnancy and the postpartum. Thyroid. 2017;27:315–89. 10.1089/thy.2016.0457 28056690

[5] Doyle K Bunch DR. Reference intervals: past, present, and future. Crit Rev Clin Lab Sci. 2023;60:466–82. 10.1080/10408363.2023.2196746 37036018

[6] Jones GRD Haeckel R Loh TP Sikaris K Streichert T Katayev A Indirect methods for reference interval determination - review and recommendations. Clin Chem Lab Med. 2018;57:20–9. 10.1515/cclm-2018-0073 29672266

[7] Thienpont LM Van Uytfanghe K Beastall G Faix JD Ieiri T Miller WG Report of the IFCC Working Group for Standardization of Thyroid Function Tests; part 1: thyroid-stimulating hormone. Clin Chem. 2010;56:902–11. 10.1373/clinchem.2009.140178 20395624

[8] Martinez-Sanchez L Marques-Garcia F Ozarda Y Blanco A Brouwer N Canalias F Big data and reference intervals: rationale, current practices, harmonization and standardization prerequisites and future perspectives of indirect determination of reference intervals using routine data. Adv Lab Med. 2020;2:9–25. 10.1515/almed-2020-0034 37359198 PMC10197285

[9] Ritchie RF Palomaki G. Selecting clinically relevant populations for reference intervals. Clin Chem Lab Med. 2004;42:702–9. 10.1515/CCLM.2004.120 15327003

[10] Protocol de seguiment de l’embaràs a Catalunya. Tercera edició. Departament de Salut;Barcelona:2018.

[11] Clinical and Laboratory Standards Institute (CLSI). Defining, Establishing, and Verifying Reference Intervals in the Clinical Laboratory; Approved Guideline − third Edition. CLSI document EP28-A3c. Wayne:CLSI;2013.

[12] Tate JR Yen T Jones GRD. Transference and validation of reference intervals. Clin Chem. 2015;61:1012–5. 10.1373/clinchem.2015.243055 26089382

[13] Farrell CL Nguyen L. Indirect reference intervals: Harnessing the power of stored laboratory data. Clin Biochem Rev. 2019;40:99–111. 10.33176/AACB-19-00022 31205377 PMC6544248

[14] Haeckel R Wosniok W Streichert T. Review of potentials and limitations of indirect approaches for estimating reference limits/intervals of quantitative procedures in laboratory medicine. J Lab Med. 2021;45:35–53. 10.1515/labmed-2020-0131

[15] Ma C Wang X Wu J Cheng X Xia L Xue F Real-world big-data studies in laboratory medicine: Current status, application, and future considerations. Clin Biochem. 2020;84:21–30. 10.1016/j.clinbiochem.2020.06.014 32652094

[16] Ma S Yu J Qin X Liu J. Current status and challenges in establishing reference intervals based on real-world data. Crit Rev Clin Lab Sci. 2023;60:427–41. 10.1080/10408363.2023.2195496 37038925

[17] Sikaris KA. Separating disease and health for indirect reference intervals. J Lab Med. 2021;45:55–68. 10.1515/labmed-2020-0157

[18] Madurga A Arbiol-Roca A Navarro-Badal MR de Basea AC-B Dot-Bach D. Strategic use of Big Data: implementing reference intervals for serum folate and serum cobalamin. Biochem Med (Zagreb). 2025;35:010705. 10.11613/BM.2025.010705 39974199 PMC11838714

[19] Stagnaro-Green A Abalovich M Alexander E Azizi F Mestman J Negro R Guidelines of the American Thyroid Association for the diagnosis and management of thyroid disease during pregnancy and postpartum. Thyroid. 2011;21:1081–125. 10.1089/thy.2011.0087 21787128 PMC3472679

[20] Yan YQ Dong ZL Dong L Wang FR Yang XM Jin XY Trimester- and method-specific reference intervals for thyroid tests in pregnant Chinese women: methodology, euthyroid definition and iodine status can influence the setting of reference intervals: Reference intervals for thyroid tests in pregnant Chinese women. Clin Endocrinol (Oxf). 2011;74:262–9. 10.1111/j.1365-2265.2010.03910.x 21044115

[21] Marwaha RK Chopra S Gopalakrishnan S Sharma B Kanwar RS Sastry A Establishment of reference range for thyroid hormones in normal pregnant Indian women. BJOG. 2008;115:602–6. 10.1111/j.1471-0528.2008.01673.x 18333941

[22] Korevaar TIM Schalekamp-Timmermans S de Rijke YB Visser WE Visser W de Muinck Keizer-Schrama SMPF Hypothyroxinemia and TPO-antibody positivity are risk factors for premature delivery: the generation R study. J Clin Endocrinol Metab. 2013;98:4382–90. 10.1210/jc.2013-2855 24037884

[23] Moon H-W Chung H-J Park C-M Hur M Yun Y-M. Establishment of trimester-specific reference intervals for thyroid hormones in Korean pregnant women. Ann Lab Med. 2015;35:198–204. 10.3343/alm.2015.35.2.198 25729721 PMC4330169

[24] Reference Intervals for Children and Adults Elecsys® Thyroid Tests TSH, FT4, FT3, T4, T3, T-Uptake, FT4-Index, Anti-TPO, Anti-Tg, Anti-TSHR, Tg, hCT cobas e analyzers. CH-6343 Rotkreuz Switzerland: Roche Diagnostics International Ltd; 2020.

[25] Rieger K Vogel M Engel C Ceglarek U Thiery J Kratzsch J Referenzintervalle für eisenabhängige Blutparameter bei Kindern und Jugendlichen: Ergebnisse einer populationsgestützten Kohortenstudie (LIFE Child). J Lab Med. 2016;40:31–41. 10.1515/labmed-2015-0093

[26] Valdés S Maldonado-Araque C Lago-Sampedro A Lillo-Muñoz JA Garcia-Fuentes E Perez-Valero V Reference values for TSH may be inadequate to define hypothyroidism in persons with morbid obesity: Di@bet.es study. Obesity (Silver Spring). 2017;25:788–93. 10.1002/oby.21796 28276648

[27] Sun T Liu X Yin C. Reference intervals for thyroid hormones in pregnant women. Endokrynol Pol. 2025;76:257–64. 10.5603/ep.100049 40586409

[28] Dorizzi RM Spiazzi G Rolli N Maltoni P Mingolla L Sgarzani C Trimester-specific reference intervals for thyroid function parameters in pregnant Caucasian women using Roche platforms: a prospective study. J Endocrinol Invest. 2023;46:2459–69. 10.1007/s40618-023-02098-0 37095269 PMC10632219

[29] Bretón I, Díaz A, Gil Á, Recio MC, Vila L, Carlos MÁ. Informe del Comité Científico de la Agencia Española de Seguridad Alimentaria y Nutrición (AESAN) en relación con la situación nutricional de la mujer en edad fértil, durante la gestación y la lactancia con respecto a la ingesta adecuada de yodo. Revista del Comité Científico de la AESAN. 2023;105-51.

[30] Torres MT Francés L Vila L Manresa JM Falguera-Puig G Prieto G Iodine nutritional status of women in their first trimester of pregnancy in Catalonia. BMC Pregnancy Childbirth. 2017;17(1):249. 10.1186/s12884-017-1423-4 28747228 PMC5530553

[31] Osinga JAJ Derakhshan A Palomaki GE Ashoor G Männistö T Maraka S TSH and FT4 Reference Intervals in Pregnancy: A Systematic Review and Individual Participant Data Meta-Analysis. J Clin Endocrinol Metab. 2022;107:2925–33. 10.1210/clinem/dgac425 35861700 PMC9516198

